# Epithelial DPP4 promotes Ang II-driven renal fibrosis by targeting ACE2 activity in the renin-angiotensin system

**DOI:** 10.7150/ijbs.106418

**Published:** 2025-06-09

**Authors:** Yingying Zhang, Ruizhi Tan, Lehao Wu, Dan Zhao, Cheng Zhong, Chenggong Luo, Jeff Yat-Fai Chung, Patrick Ming-Kuen Tang, Chen Yu

**Affiliations:** 1 Department of Nephrology, Tongji Hospital, Tongji University School of Medicine, Shanghai, China.; 2 Research Center of Combined Traditional Chinese and Western Medicine, Affiliated Traditional Medicine Hospital, Southwest Medical University, Luzhou, Sichuan, China.; 3 Department of Anatomical and Cellular Pathology, Peter Hung Pain Research Institute, Li Ka Shing Institute of Health Sciences, State Key Laboratory of Translational Oncology, Prince of Wales Hospital, The Chinese University of Hong Kong.

**Keywords:** Renal fibrosis, Dipeptidyl peptidase-IV, Renin-angiotensin system

## Abstract

Renal fibrosis is a characteristic of the progression of various chronic kidney diseases (CKD) to end-stage renal disease (ESRD). The renin-angiotensin system (RAS) is key to renal pathology. A better understanding of its regulatory mechanisms at the molecular level may lead to solutions for clinical CKD. Interestingly, our cohort study observed a positive correlation between epithelial dipeptidyl peptidase-IV (DPP4) levels and clinical CKD progression. Consistently, DPP4 was significantly increased in the unilateral ureteral obstruction (UUO) injured kidney *in vivo* and human epithelial kidney HK-2 cells under Ang II stimulation *in vitro*. Unexpectedly, kidney-specific deletion of DPP4 effectively ameliorated UUO and ischemia/reperfusion (I/R)-driven renal fibrosis *in vivo*. Mechanistically, we reveal that DPP4 serves as a novel inhibitor of the Ang(1-7)/MasR axis and an inducer of the AT1R axis by directly binding to ACE2 at the protein level. More importantly, targeting DPP4 with pharmaceutical inhibitor linagliptin effectively restored anti-fibrotic pathway of RAS, thereby blocking the CKD progression of I/R-injured kidney *in vivo*. Therefore, epithelial DPP4 may represent a precise therapeutic target to enhance the anti-fibrotic activity of RAS for CKD treatment in the clinic.

## Introduction

Renal fibrosis is the critical pathway leading to end-stage kidney disease; however, its effective treatment is still an unmet need in clinics. Increasing evidence suggested that renin-angiotensin system (RAS) plays a central role in renal fibrosis 1. RAS encompasses two distinct pathways: the classical angiotensin II (Ang II)/ angiotensin II type 1 receptor (AT1R) axis, which promotes renal interstitial fibrosis, and the angiotensin converting enzyme 2 (ACE2)/ angiotensin (1-7) (Ang(1-7))/ G-protein-coupled Mas receptor (MasR) axis, which counteracts this process and delays renal fibrosis. ACE2, a key protein within the ACE2/Ang(1-7)/MasR pathway, exerts its biological function through enzymatic activity 2. It can cleave Ang II and inhibit its binding to AT1R; Ang II, after being cleaved, generates Ang (1-7), which acts on MasR and exhibits anti-fibrotic effects 3. Therefore, ACE2 activity plays a crucial role in maintaining balance between the ACE2/Ang(1-7)/MasR and Ang II/AT1R axis. Nevertheless, an effective strategy for rebalancing the RAS in chronic kidney disease is still unavailable worldwide.

Dipeptidyl peptidase-IV (DPP4, also named CD26) is originally found to be highly expressed on adipocytes and hepatocytes due to elevated glucose levels and obesity 4. It is responsible for the homeostasis of blood glucose level by regulating the degradation of glucagon-like peptide 1 (GLP-1) and glucose-dependent insulinotropic polypeptide (GIP). Moreover, DPP4 is further found to be expressed in various organs, including the liver, pancreas, adipose tissue, intestines, as well as kidneys 5. DPP4 is highly expressed in the renal endothelial cells, tubular epithelial cells (specifically S1-S3 segments) and collecting ducts during the progression of diabetic nephropathy 6. Interestingly, DPP4 targeted therapy unexpectedly showed reno-protective effect in clinics, where a subgroup analysis of CARMELINA study detected that a DPP4 inhibitor (DPP4i) linagliptin exhibits protective effect on patients with moderate renal dysfunction (CKD stage 3) 7. It is also reported that systematic inhibition of DPP4 effectively suppresses the progression of c-reactive protein driven diabetic nephropathy in db/db mice, highlighting its potential reno-protective effect beyond glycemic control 8. Nevertheless, the roles of DPP4 in the development of renal fibrosis are still largely unexplored.

Here, we observed that DPP4 is dominantly expressed on the renal epithelial cells in kidneys of CKD patients and experimental models' unilateral ureteral obstruction (UUO) and ischemia/reperfusion injury (I/R), which is positively correlated with the renal fibrosis and failure in clinics. We demonstrated that kidney-specific deletion of DPP4 effectively suppresses renal fibrosis with UUO and I/R *in vivo*. Importantly, we unexpectedly identified that DPP4 interacts with ACE2 and inhibits its enzymatic activity at protein level, therefore up-regulating the profibrotic pathway of RAS *in vitro* and *in vivo*. More encouragingly, we demonstrated that treatment with DPP4i, linagliptin, a Food and Drug Administration (FDA) approved drug, not only suppresses the AT1R but also enhances MasR expressions in the UUO- and I/R-injured kidneys, representing an effective strategy for rebalancing the renal RAS. Our findings may serve as important rationales for the development of DPP4i as a therapeutic strategy for RAS-driven kidney fibrosis in clinics.

## Materials and Methods

### Human kidney tissues

The human kidney tissues used in this study were collected from the Department of Nephrology, Tongji Hospital, Shanghai. Kidney tissues were obtained from patients with IgA nephropathy (IgAN) without diabetes and had been evaluated by biopsy procedures. The use of all patient biopsy samples was approved by the Human Subjects Committee of Tongji Hospital (No. [K-W-2020-009]). All patients gave written informed consent before entering the study.

### Animals

To generate compound mice Dpp4fl/fl; Cdh16-Cre, Dpp4fl/fl mice were crossed with Cadherin 16-Cre mice (Tg (Cdh16-cre)91Igr/J), both of mice were purchased from Shanghai Saiye company. All animals were kept in the animal room of Tongji Hospital affiliated with Tongji University, group-housed under constant temperature (23±2 ℃) and humidity (40-60%), and provided water *ad libitum*. All animal procedures and protocols were approved by the Ethics Committee of Tongji University School of Medicine. The establishment of two mouse models of CKD was achieved through the induction of ischemia/reperfusion injury (I/R) or unilateral ureteral occlusion (UUO) 9.

In the renal I/R model, after flank incision, the left renal pedicle was clamped for 30 mins using nontraumatic microvascular clamps at 37 °C. Then, the clamps were released for reperfusion after ischemia. Twenty-eight days after ischemia, I/R group mice were sacrificed. UUO) was induced by ligating the left ureter. UUO group mice were sacrificed on day 7 after UUO surgery, and the left kidneys were collected. Mice were treated with linagliptin, 3 mg/kg/day for each mouse, after ischemia, and all mice were sacrificed at day 28 via injection of ketamine/xylene.

### Cell culture and treatment

HK2 cells were cultured in Dulbecco's modified Eagle's medium (DMEM) (Gibco, #10569044) supplemented with 10% fetal bovine serum (FBS) (Gibco, #10100147), as described previously 8. Cells were treated with TGF-β1 (R&D Systems, USA) or Ang II (MCE, HY13948, USA). Whole-cell lysates were prepared and subjected to western blot analyses or real-time PCR. We use DPP4i (Boehringer Inghelheim, Germany) to downregulate DPP4 expression, and BAY11-7082 (MilliporeSigma. Germany) to inhibit the phosphorylation of NF-ҡB, respectively, for 2 h before addition of Ang II for 24 h. All of the experiments were repeated independently at least three times. The DPP4-targeted siRNA (the used sequences were listed in supplementary material) were transfected into HK2 cells with Dharmacon DharmaFECT (T-2001-xx, Horizon) before Ang II treatment following the manufacturer's instructions 10.

### Western blotting

HK2 cells and kidney cortex were lysed in radioimmunoprecipitation assay (RIPA) lysis buffer. Western blot analysis was performed as described previously. Antibodies used in the current study include DPP4 (sc-52642; Santa Cruz), ACE2 (sc-390851; Santa Cruz), Mas1 (#AAR-013; Alomone Labs and K000494; Solarbio), AT1R (sc-515884; Santa Cruz)^11^, and other antibodies involved, such as fibronectin (Fn), α-smooth muscle actin (α-SMA), phospho-NF-ҡB/p65, NF-ҡB/p65, and β-actin, were described previously^8^. Then IRDye800-conjugated secondary antibodies (CST) were used as secondary antibodies. Signals were detected using the LiCor/Odyssey infrared image system (LI-COR Biosciences, Lincoln, NE, USA), Densitometry was performed using Quantity One software.

### Reverse transcription and quantitative real-time PCR

The RNAs were extracted from cells and kidney tissues using TRIzol reagent (Invitrogen, Carlsbad, CA) as previously described. The primers used in this study targeted mouse and/or human DPP4 and ACE2 were listed in Table S2^8^, ^11^.

### Histology and immunofluorescent staining

Paraffin-embedded kidney sections (4-μm thickness) were prepared according to a standard procedure. Masson trichrome staining and Sirius staining were performed by using a standard protocol. For immunofluorescence staining, kidney cryosections were fixed with 4% paraformaldehyde for 30 minutes at room temperature and then blocked with 5% goat serum. The primary antibodies were used the same as western blot assay. Either FITC or Texas Red were used as secondary antibodies. Fibrotic area was quantified by Masson trichrome staining, where 20 glomeruli were randomly selected from each section, and positive signals within the selected area were highlighted, measured. The percentage of positive staining areas of Fn and α-SMA expression was quantified using the same software in 10 consecutive fields as previously described 12.

### Chromatin immunoprecipitation analysis

The Chromatin immunoprecipitation (ChIP) assay was conducted using a transcription factor ChIP kit (CST #9003) in accordance with the manufacturer's instructions. HK2 cells were treated with or without Ang II for the indicated times. Immunoprecipitation was performed with the antibody against NF-ҡB (#8242; Cell Signaling) or a normal IgG as negative control. Precipitated DNA fragments were detected by PCR using a specific primer of the promoter region of DPP4, showing in Table S1.

### Immunoprecipitation

HK2 cells were extracted in ice-cold RIPA lysis buffer as previously described. Primary antibodies, including DPP4 (sc-52642; Santa Cruz) and ACE2 (sc-390851; Santa Cruz), were added into the equal lysis buffer supernatant. After incubating overnight at 4°C, the immune complex is captured, or precipitated, on a beaded support to which an antibody-binding protein is immobilized (such as protein A or G). Finally, the immune complexes were washed with lysis buffer three times and then boiled in SDS sample buffer for 5 min, followed by western blotting as described above 8.

### Truncation domain construction

Full-length DPP4 and truncated DPP4 were cloned into the pcDNA3.1 vector (Invitrogen, USA) and subsequently verified by double-strand DNA sequencing. Full-length DPP4 and truncated DPP4 (amino acids sequence: 1-766, 1-492, 1-290, 1-49) clones were generated from human DPP4 (Gene bank ID: 1803). Primers for constructing various clones of truncated DPP4 are shown in Table S3. 293T cells (~60% confluence) were transiently transfected by 5-10 µg cDNAs carrying either full length DPP4(or other truncated domain of DPP4) or 3 µg GFP cDNAs using Lipofectamine 3000 reagent (Thermo Fisher Scientific, USA). All cells were kept in a humidified incubator with 5% CO2 at 37°C. All the cells were harvested after 48 h transfection 13.

### GST pull-down assays

GST-DPP4 fusion protein-expressing constructs were generated in a pGEX-4T-1 vector by cloning fragments of human DPP4 complementary DNA (cDNA) (GenScript) into the Bam HI and ECQRI sites via polymerase chain reaction (PCR). GST-DPP4 constructs (Extracellular Region) were transformed to BL21 *E.coli* and induced using isopropyl-β-d-thiogalactopyranoside to a final concentration of 1mM. The GST protein was purified from E. coli bacteria by using glutathione-agarose beads (Novagen), and expression of the constructs was analyzed on SDS-PAGE gels stained with Coomassie blue. In parallel, the purified GST-DPP4 fusion proteins were incubated with 1 μg of ACE2 protein (ab277810, abcam). Following washing using PBST, the protein complexes were run on SDS-PAGE gels. Immunoblot analyses were performed using a mouse anti-ACE2 antibody 13.

### Protein-protein docking test

HACE2 and hDPP4 protein structures were constructed by Uniport. A full-length structural model of hACE2 was constructed using Q9BYF1 for homology modeling, with amino acids 20-740 modeled based on the template from protein data bank (PDB) (6m18). *De novo* modeling provided structural coordinate information for amino acids 1-19 and 741-805. Similarly, a full-length structural model of hDPP4 was constructed using P27487 for homology modeling. The template used for homology modeling is PDB :1wcy, applied to amino acids 38-766 and 1-37. The protein-protein docking can be conducted using a global docking approach based on fast Fourier transform and energy scoring function. Root mean square fluctuations (RMSFs) were determined to examine the allosteric situation of local sites. The test was conducted by Shanghai Yuyibiotech Inc 14, 15.

### Renal function measurement

Levels of serum creatinine were tested accordingly with the enzymatic method (Stanbio Laboratories, TX, USA) as in a previous study 16. Levels of serum blood urea nitrogen (BUN) were tested accordingly with the enzymatic method (Quantichrom, Cat DIUR-100) as in a previous study.

### ACE2 enzymatic activity assay

Activity of ACE2 in HK2 cells was measured by ACE2 activity assay kit (Fluorometric) (Abcam, ab273297), which utilizes the ability of an active ACE2 to cleave a synthetic MCA based peptide substrate to release a free fluorophore 17.

### ELISA assay

Kidney Ang II (Elabscience, E-EL-M2612) and Ang 1-7 (Elabscience, E-EL-M2681) levels were measured by ELISA assay. Kidney tissues were collected and immediately immersed in a five-fold volume of cold PBS. Following centrifugation, the supernatants were analyzed.

### Statistical analysis

All values examined are shown as the mean ± SEM for each group. Intergroup comparisons were made using one-way analysis of variance. Student's t-test was used to analyze data between two groups. The Mann-Whitney test was used for the two groups of data that did not conform to the normal distribution. Spearman's correlation analysis was used to assess the correlation. In all tests, p < 0.05 was considered a statistically significant difference between the mean values. GraphPad prism 9.0 software was used for the statistical analyses.

## Results

### DPP4 is mainly expressed in the epithelial cells of fibrosing kidney

**Figure 1:**
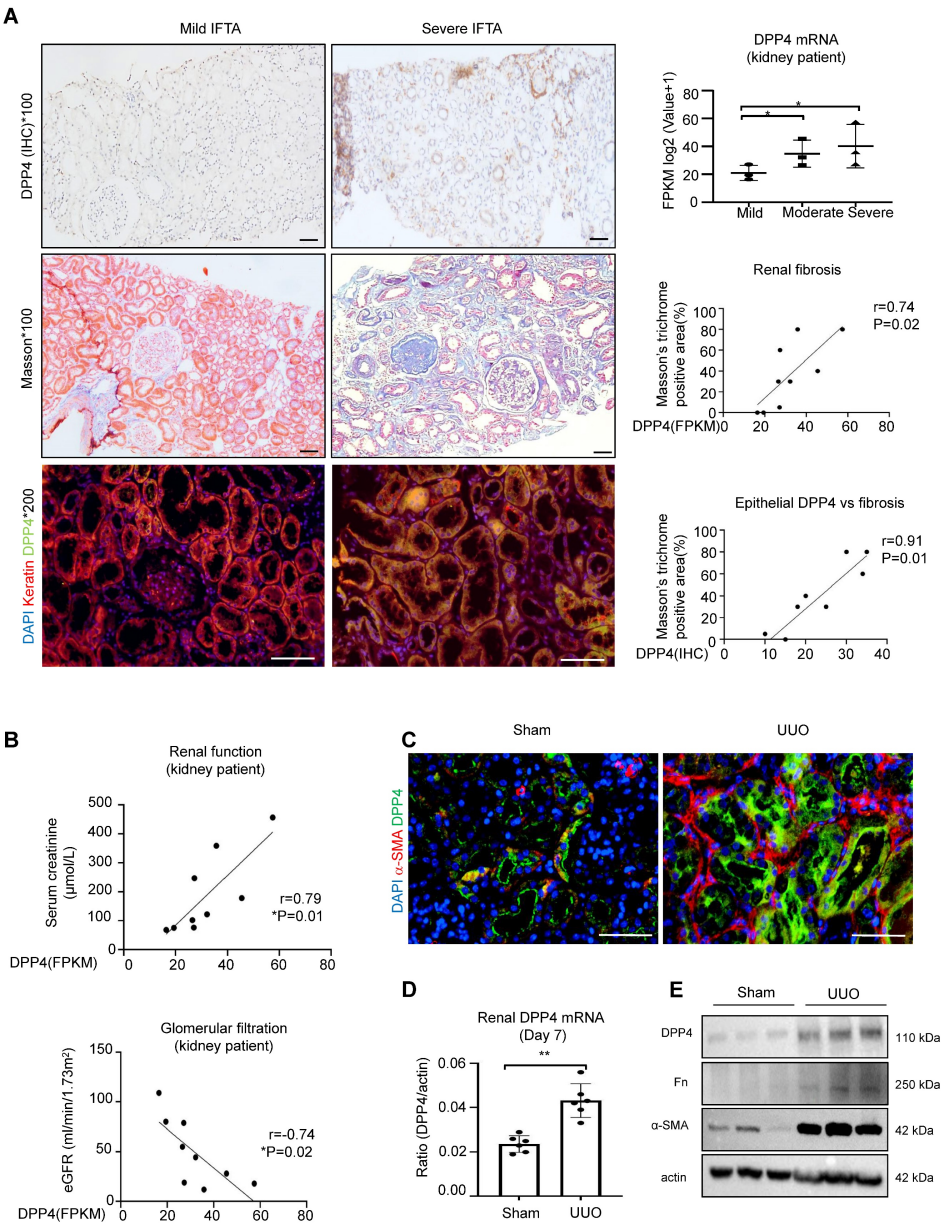
** DPP4 expression increased in kidney of patients with IgAN and UUO injured models.** (A) IHC staining for DPP4, Masson's trichrome staining and Immunofluorescence (IF) staining for co-localization of DPP4 (green) and Keratin (red) from IgAN patients. Fragments Per Kilobase of transcript per Million (FPKM) of DPP4 gene expression in the kidneys of IgA patients. The correlation between positive areas of DPP4 and FPKM of DPP4 gene expression and Masson's positive area was estimated in all IgA patients (n = 9). (B) The correlation between DPP4 gene expression and serum creatinine levels and estimated glomerular filtration rate (eGFR) in all IgA patients. (C) IF detected DPP4 (green), α-SMA (red) and nuclei (DAPI, blue) in the sham and unilateral ureteral occlusion (UUO)-induced mice kidneys. n=6 (D-E) Real-time PCR (D) and western blot analysis (E) showed DPP4 expression from the sham and (UUO) kidneys. n=6 *In vitro* experiments were analyzed from three experiments. All statistical data are represented as mean ± SEM in A and D. *p < 0.05, **p < 0.01. Bar=50μm.

To elucidate the clinical importance of DPP4 in renal fibrosis, we examine its expression on patients with IgA nephropathy (IgAN). Interestingly, we found that the DPP4 is gradually increased in the diseased kidneys associated with the progression of IgA and renal fibrosis in the patients, where DPP4 is predominately expressed on the renal epithelial cells showing by the epithelial specific marker keratin (Figure 1A). We further detected that renal transcription level of DPP4 is positively correlated to the renal failure in the IgA patients according to their changes of serum creatinine and glomerular filtration rate (Figure 1B), where an up-regulated KEGG pathway is related to DPP4 (Supplementary Figure 1A). By screening the online single-cell database, we identified a significant upregulation of DPP4 in the proximal renal tubular epithelium (S1-S3 segment) of mice in the UUO-injured kidneys compared to the healthy adult mouse kidney (CCA) (Supplementary Figure 1B). Additionally, we found a dramatic up-regulation of DPP4 expression in the UUO-injured kidney in mice at both protein and mRNA levels associated with the expression of renal fibrosis markers fibronectin (Fn) and α smooth muscle actin (α-SMA) *in vivo* (Figure 1C-E). These findings suggest a potential role of renal epithelial DPP4 in the progression of renal fibrosis.

### Ang II / NF-ҡB signaling regulates DPP4 transcription in renal epithelial cells

**Figure 2:**
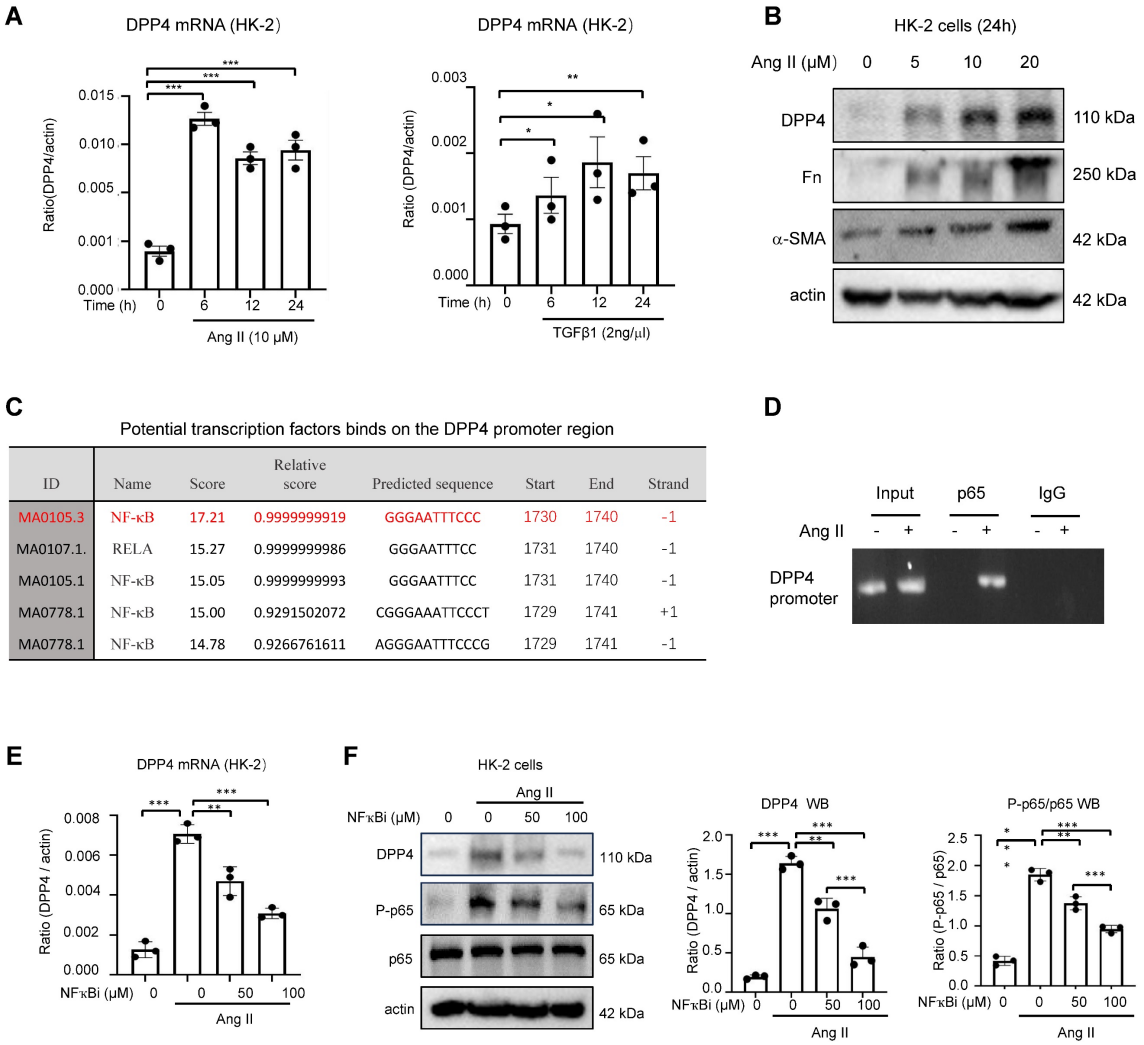
** Ang II / NF-ҡB signaling regulates DPP4 transcription in renal epithelial cells.** (A) Real-time PCR showed that DPP4 expression peak at 6 hours after Ang II (10μM) treatment, whereas the peak of DPP4 expression was observed at 12 hours after TGF-β1 (2ng/ml) treatment in HK2 cells. (B) Western blot analysis revealed a dose-dependent increase in DPP4 expression induced by Ang II in HK2 cells after 24 hours, which is consistent with the increased expression of α-SMA and fibronectin (Fn). (C) The predicted binding site of NF-ҡB on the evolutionarily conserved region of DPP4 by JASPER browser. (D) ChIP assay shows that NF-ҡB physically binds DPP4 promoter in response to Ang II (10μM) in HK-2 cells. (E, F) Real-time PCR (E) and western blot (F) analysis show the addition of NF-ҡB inhibitor BAY11-7085 blocks the activation of Ang II-induced DPP4 expression in HK-2 cells. Each bar represents the mean ± SEM of at least three independent experiments in A, E and F. *p < 0.05, **p < 0.01, ***p < 0.001.

As RAS is well-documented for driving the development of renal fibrosis in IgAN^18^, we examined its potential role in DPP4 regulation on the human renal epithelial cell line HK-2 *in vitro*. Surprisingly, we observed a stronger effect of Ang II on inducing DPP4 transcription in HK-2 cells after 6 hours post-treatment compared to the fibrotic effector TGF-β1 (Figure 2A). We confirmed that Ang II can dose-dependently trigger DPP4 expression in HK-2 cells associated with the fibrotic signature genes Fn and α-SMA *in vitro* (Figure 2B). Furthermore, bioinformatic analysis suggested strong binding of an inflammatory transcription factor NF-ҡB on the promoter region of the DPP4 genomic sequence (Figure 2C), which was experimentally confirmed on the Ang II stimulated HK-2 cells by ChIP-PCR assay *in vitro* (Figure 2D). Moreover, application of NF-ҡB inhibitor (NF-ҡBi) markedly suppressed the Ang II driven DPP4 expression on the HK-2 cells at both mRNA and protein levels (Figure 2E, 2F). Our findings clearly demonstrate the significant role of the Ang-II/NF-κB pathway in regulating DPP4 at the transcriptional level.

### DPP4 inhibition blocks Ang II driven fibrotic signatures *in vitro*

As the role of DPP4 in the Ang II mediated renal fibrosis is unknown, we examined the changes of fibrotic gene expression in the HK-2 cells with genetical and pharmaceutical inhibition of DPP4 *in vitro*.

**Figure 3:**
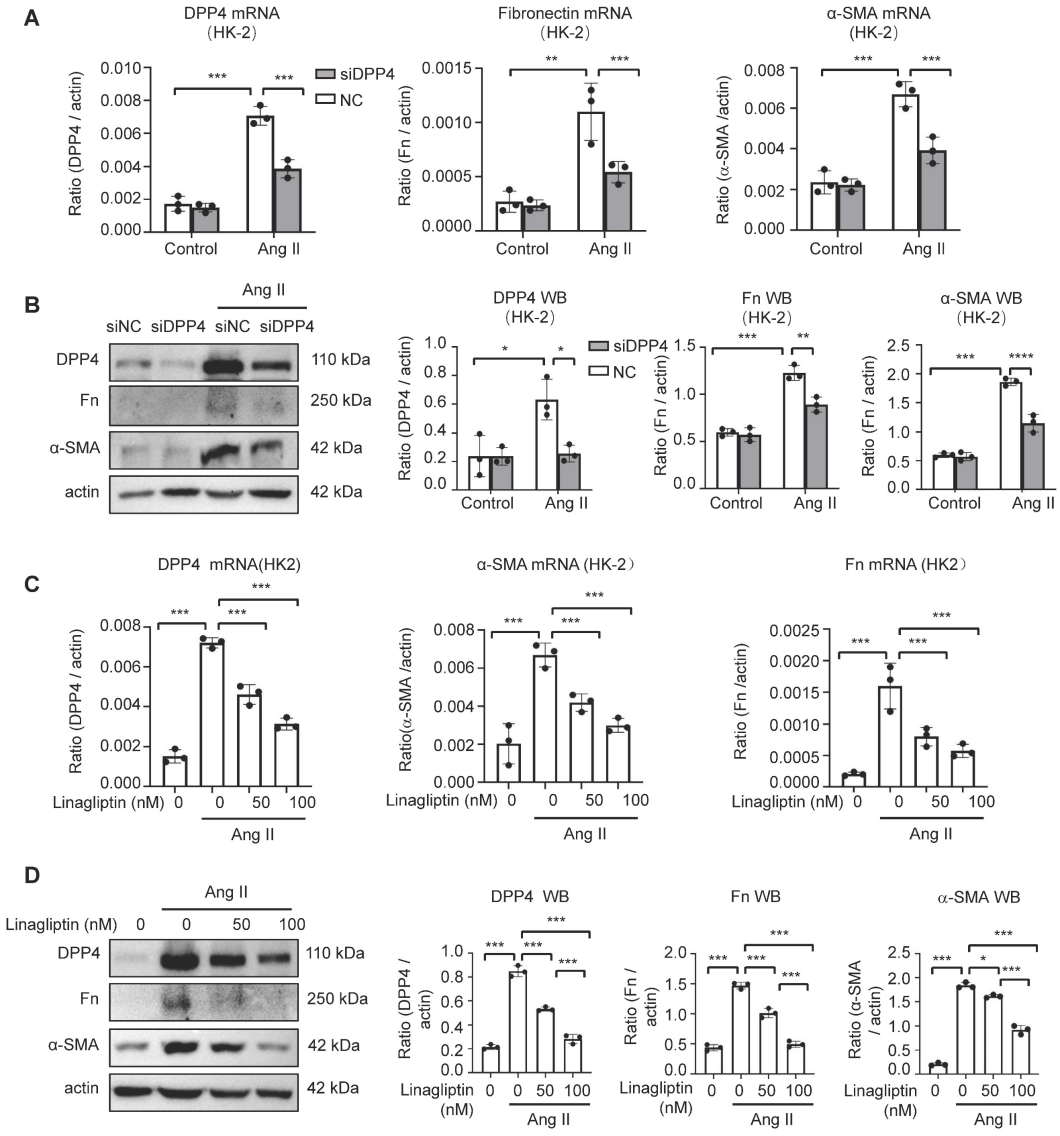
** Knockdown or inhibition of DPP4 alleviated Ang II-induced fibrosis formation, respectively, *in vitro*.** (A) Real-time PCR shows DPP4 expression after knockdown of DPP4 compared to the control siRNA (siNC) group in HK-2 cells upon Ang II-induced stimulation. Real-time PCR shows Fn and α-SMA expression after knockdown of DPP4 in HK-2 cells. (B) Western blot analysis shows that knockdown of DPP4 decreased the protein expression of Fn and α-SMA. (C, D) Real-time PCR (C) and western blot analysis (D) show inhibition of DPP4 decreased the protein and mRNA expression levels of Fn nd α-SMA in Ang II -treated HK2 cells at 24 hours. Data represent the mean ± SEM of at least three independent experiments. *p < 0.05, **p < 0.01, ***p < 0.001.

Interestingly, genetic silencing of DPP4 (siDPP4) markedly suppressed the Ang II driven fibrotic signatures in the HK-2 cells, showing by the significant reduction of Fn and α-SMA expression at mRNA and protein levels *in vitro* (Figure 3A, B). Moreover, by using a Food and Drug Administration (FDA) approved DPP4 inhibitor linagliptin 19, we demonstrated that DPP4 inhibition can dose-dependently suppress the fibrotic signature on the Ang II stimulated HK-2 cells *in vitro* (Figure 3C, D), uncovering its clinical potential for targeting the Ang II driven renal fibrosis.

### Tubular epithelial specific knockout of DPP4 attenuated renal fibrosis in UUO mice

**Figure 4:**
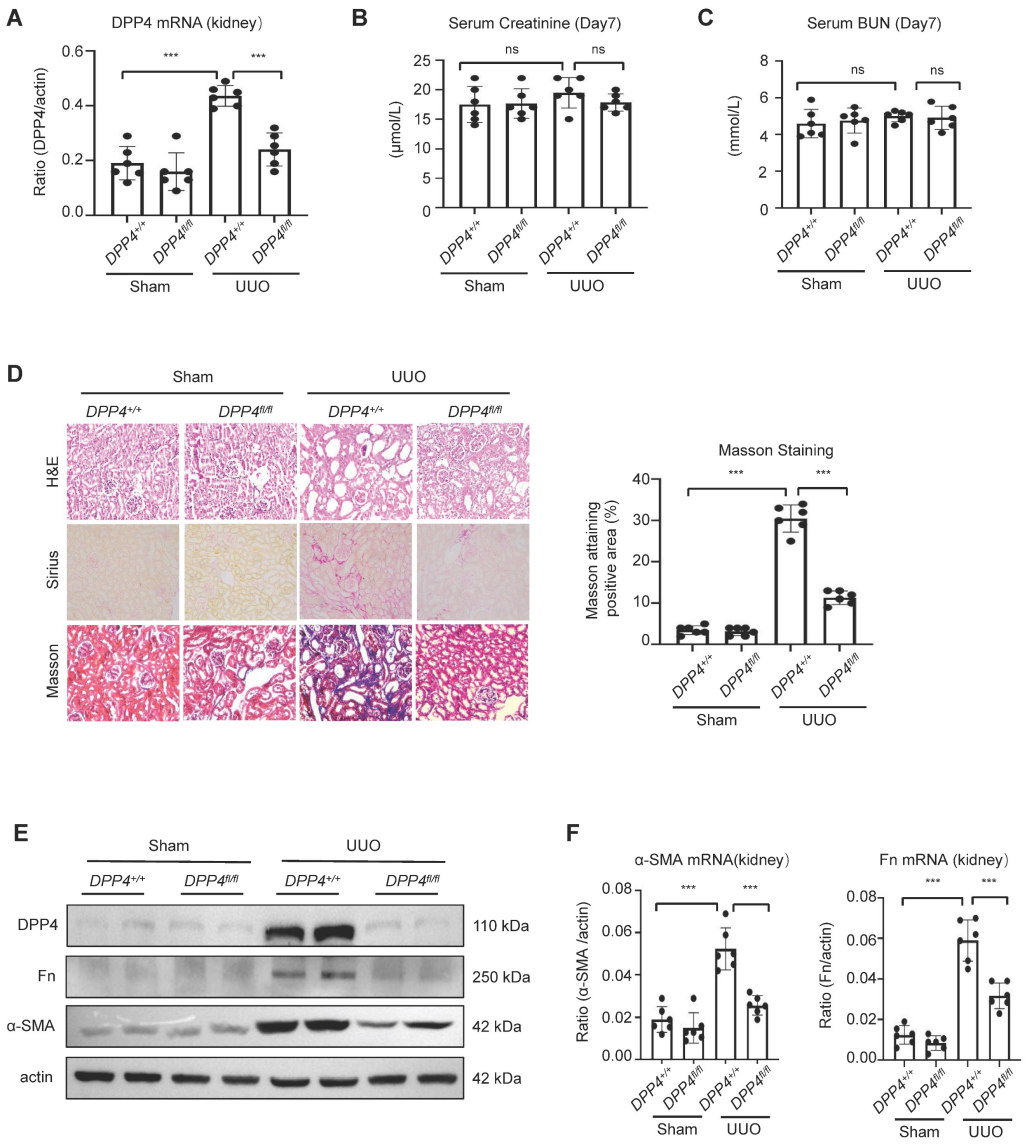
** DPP4 conditional knockout protects against renal dysfunction and suppresses renal fibrosis in UUO-induced mouse model.** (A) Real-time PCR shows mRNA expression levels of Dpp4, Fn and α-SMA in mice kidneys. (B) Serum creatinine of the mice in different groups. (C) Serum BUN of the mice in different groups. (D) Representative image showed the Hematoxylin and eosin (HE), Sirius staining and Masson trichrome staining of the kidneys, and quantification analysis of fibrotic area calculated by Masson trichrome staining. (E) Western blot of DPP4, Fn and α-SMA expression in the kidneys. (F) Real-time PCR of Fn and α-SMA expression in the kidneys of different groups. Data represent the mean ± SEM. n=8. *p < 0.05, ***p< 0.001. n.s., statistically not significant.

To investigate the functional role of DPP4 *in vivo*, we generated DPP4 conditional knockout Dpp4 fl/fl; Ksp-Cre (Dpp4 fl/fl) mice, which had kidney-specific cadherin (Ksp-cadherin) driving Cre expression 20 (Supplementary Figure 2A). Next, the Dpp4fl/fl; Ksp-Cre mice were subjected for a classic renal fibrosis model UUO. We found a significant increase in DPP4 expression in the tubular epithelial cells of UUO-injured kidneys in the Dpp4+/+; Ksp-Cre (Dpp4+/+) mice as evidenced by co-localization with lotus tetragonolobus lectin (LTL), a marker of proximal tubules. In contrast, DPP4 expression was abolished in the epithelial cells of Dpp4fl/fl; Ksp-Cre mice, regardless of UUO injury (Supplementary Figure 2B). qRT-PCR analysis revealed that Dpp4 mRNA levels were significantly elevated in the kidney of Dpp4+/+; Ksp-Cre mice following UUO injury, whereas it remained at baseline levels in the kidneys of Dpp4 fl/fl; Ksp-Cre mice subjected to UUO injury (Figure 4A). We found that the serum creatinine (Scr) and urea nitrogen (BUN) levels didn't increase following the UUO injured both in Dpp4 fl/fl; Ksp-Cre and Dpp4+/+; Ksp-Cre mice, we proceeded to evaluate fibrotic changes within renal tissues (Figure 4B, 4C). Hematoxylin and eosin (H&E) showed that the renal tubular injury induced by UUO treatment were also attenuated in Dpp4 fl/fl; Ksp-Cre mice (Figure 4D). As shown by Sirius staining and Masson trichrome staining, UUO injury mice had more positive areas of interstitial fibrosis than sham group, and Dpp4 fl/fl; Ksp-Cre reduced the increase of positive areas of interstitial fibrosis in UUO injury mice (Figure 4D). In order to investigate the effects of DPP4 on extracellular matrix precipitation after UUO injury, the expressions of α-SMA and Fn were detected by immunofluorescence analysis and western blot analysis. Results showed that the Fn and α-SMA expression were significantly elevated in kidneys of Dpp4+/+; Ksp-Cre mice after UUO treatment, and these changes were blunted in Dpp4fl/fl; Ksp-Cre mice kidneys (Figure 4E and Supplementary Figure 2C). Similar changes in Fn and α-SMA were confirmed by evaluating the mRNA levels (Figure 4F).

### Tubular epithelial specific knockout of DPP4 attenuated renal fibrosis in I/R mice

**Figure 5:**
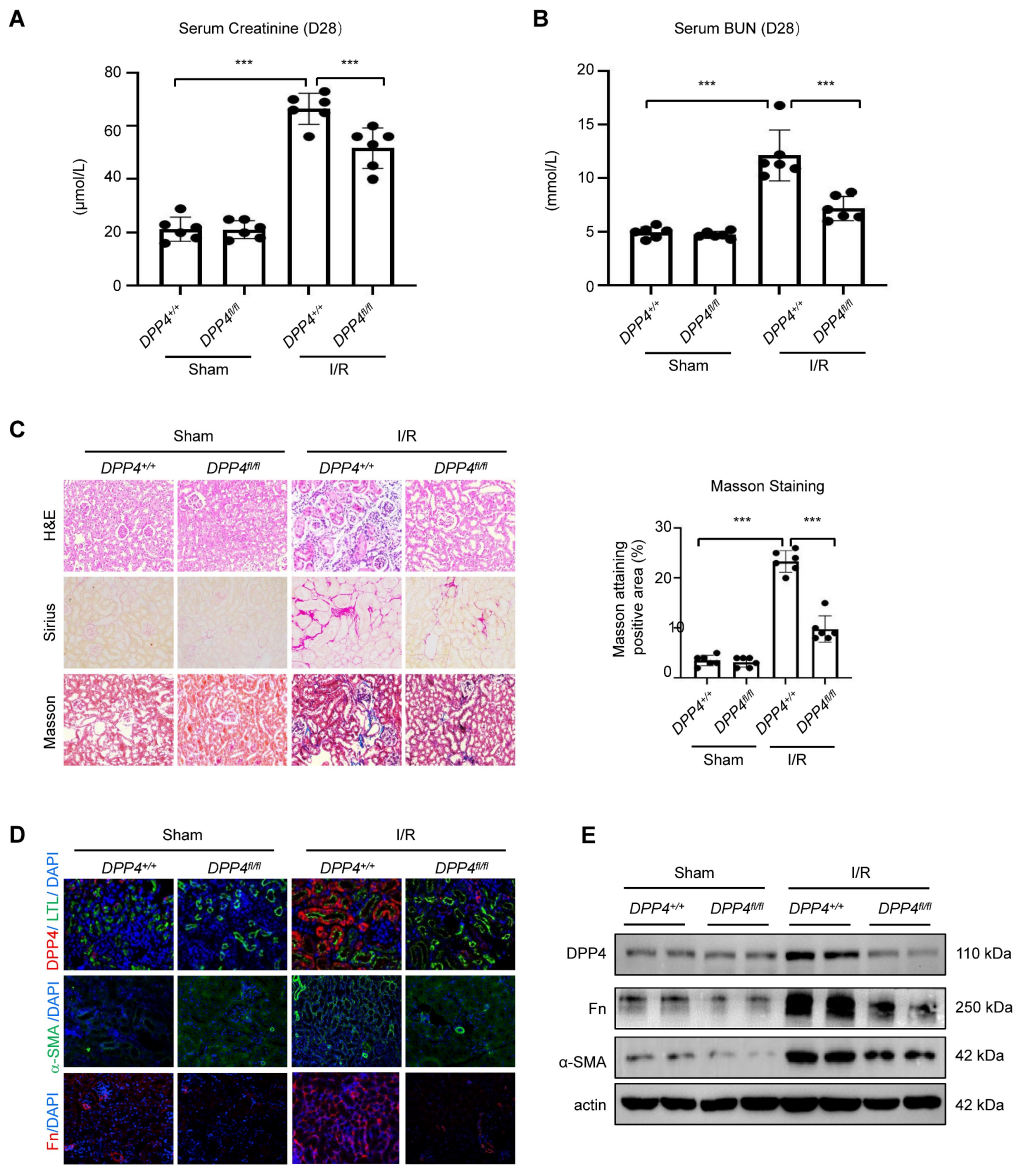
** DPP4 conditional knockout protects against renal dysfunction and suppresses renal fibrosis in I/R-induced mouse model.** (A, B) Serum creatinine (A) and BUN (B) of the mice in different groups. (C) Representative image showed the Hematoxylin and eosin (H&E), Sirius staining and Masson trichrome staining of the kidneys, and quantification analysis of fibrotic area calculated by Masson trichrome staining. (D) Immunofluorescent (IF) analysis of co-staining of DPP4 (Red) and LTL (Green), IF staining of Fn and α-SMA in kidneys. (E) Western blot of DPP4, Fn and α-SMA expression in the kidneys. Data were expressed as means ± SEM. n=8. ***p < 0.001.

To further demonstrate the role of DPP4 under fibrotic condition, we established another fibrotic model, the I/R induced animal model. We found that deletion of epithelial DPP4 effectively protected against I/R-induced kidney dysfunction in Dpp4 fl/fl; Ksp-Cre (Dpp4fl/fl) mice, as evidenced by reduced serum creatinine and BUN levels (Figure 5A and 5B). We found a significant increase in DPP4 expression in the tubular epithelial cells of I/R-injured kidneys in the Dpp4+/+; Ksp-Cre (Dpp4+/+) mice as evidenced by co-localization with LTL and DPP4. In contrast, DPP4 expression was abolished in the epithelial cells of Dpp4fl/flfl; Ksp-Cre mice, regardless of I/R injury (Figure 5D). HE, Sirius red staining, and Masson trichrome demonstrated that I/R injury induced significant renal tubular damage and interstitial extracellular matrix (ECM) deposition in the kidneys of Dpp4+/+; Ksp-Cre (Dpp4+/+) mice compared to those of Dpp4 fl/fl; Ksp-Cre mice (Figure 5C). Immunofluorescence staining analysis and western blot analysis showed that the increase expression of fibronectin (Fn) and α-SMA was alleviated in I/R induced Dpp4 fl/fl; Ksp-Cre mice when compared with Dpp4+/+; Ksp-Cre mice (Figure 5D and 5E). These results suggested that TEC-specific knockout of Dpp4 attenuated UUO- and I/R-induced renal dysfunction, tubular injury and interstitial ECM deposition.

### Ang II triggers DPP4 interaction with ACE2 in renal epithelial cells *in vitro*

**Figure 6:**
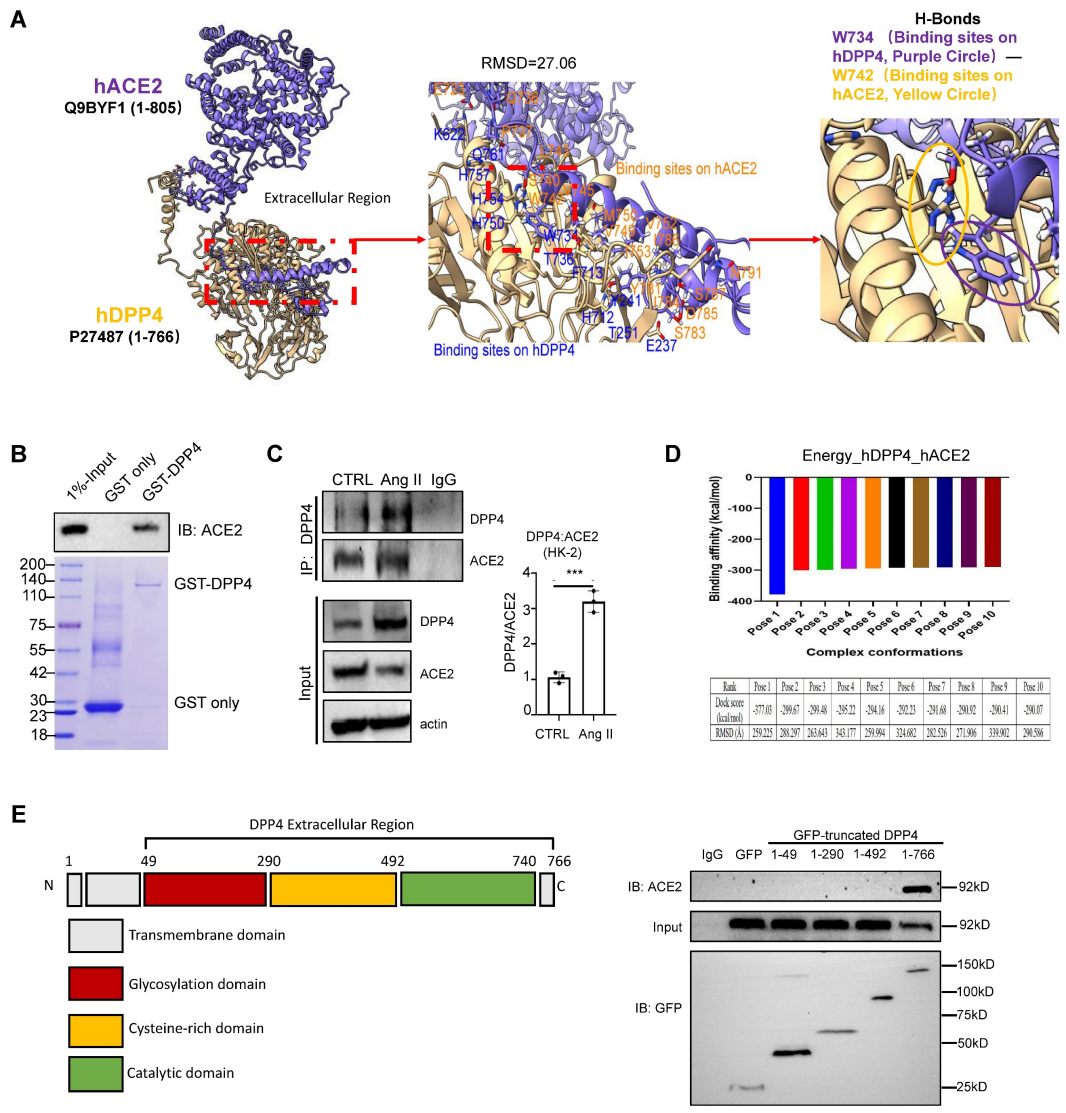
** DPP4 interacts with ACE2.** (A) Uniprot acquired the complete structural models of hDPP4 (yellow) and hACE2 (purple). Interactive interface assay to identify specific binding sites between hACE2 and hDPP4. A H-bond between DPP4 (W734) and ACE2 (W742) is shown in the enlarged panel. (B) GST pull-down using bacteria-expressed GST-LSD1 and purified ACE2 proteins. (C) Co-immunoprecipitation (Co-IP) assay demonstrates that Ang II triggers the binding of DPP4 to ACE2 in HK-2 cells at 24 h after stimulation. Data represent the mean ± SEM of at least three independent experiments. ***p < 0.001. (D) Fourier transform and energy scoring are used to calculate the optimal conformation of DPP4 and ACE2 interaction. The calculated binding energy for Pose 1 is -377.03 kcal/mol, while the root-mean-square deviation (RMSD) of this pose measures 259.225 Ångström (Å). (E) GFP-tag truncated construction of DPP4 establishment, and Co-IP assay demonstrates that ACE2 binds to the Catalytic domain of DPP4.

As ACE2 is the key regulator of RAS, we examined its potential interaction with DDP4 by proteomic analysis 2. Protein-protein interaction (PPI) network predictions suggest that, in addition to its known interactions with glucagon-like peptide-1 receptor (GLP1R) and gastric inhibitory polypeptide (GIP), DPP4 also interacts with ACE2 (Supplementary Figure 3A). Immunofluorescence staining revealed that both DPP4 and ACE2 were expressed in the epithelial cells of the proximal renal tubules (Supplementary Figure 3B). Additionally, by conducting molecular docking analysis 15, we found a strong physical binding between ACE2 and DPP4 proteins at molecular level (Figure 6A). The direct interaction between DPP4 and ACE2 was experimentally confirmed by GST pulldown assays at protein level (Figure 6B), we found that formation of DPP4:ACE2 can be markedly enhanced in the HK-2 cells by Ang II stimulation *in vitro* (Figure 6C). In addition, according to the result of binding affinity (pose 1, Figure 6D), catalytic domain of DPP4 is predicted to be the most preferred site for interacting with ACE2 at the protein level (aa493-740 in Figure 6E) which is experimentally confirmed by pull-down assay with GFP-expressing DPP4 truncated protein (Figure 6E). It is reported that the C-terminal of DPP4 is the catalytic domain, a cysteine-rich area, and a large glycosylated region linked by a flexible stalk to the transmembrane segment. The active site, Ser 630, is flanked by the classic serine peptidase motif Gly-Trp-Ser630-Tyr-Gly-Gly-Tyr-Val 5,21. Our findings provided the evidence of direct interaction between DPP4 and ACE2 for regulating the RAS under high Ang II condition at its catalytic domain.

### DPP4 promotes renal fibrosis via disrupting ACE2/Ang (1-7)/MasR and Ang II/AT1R axis of RAS

**Figure 7:**
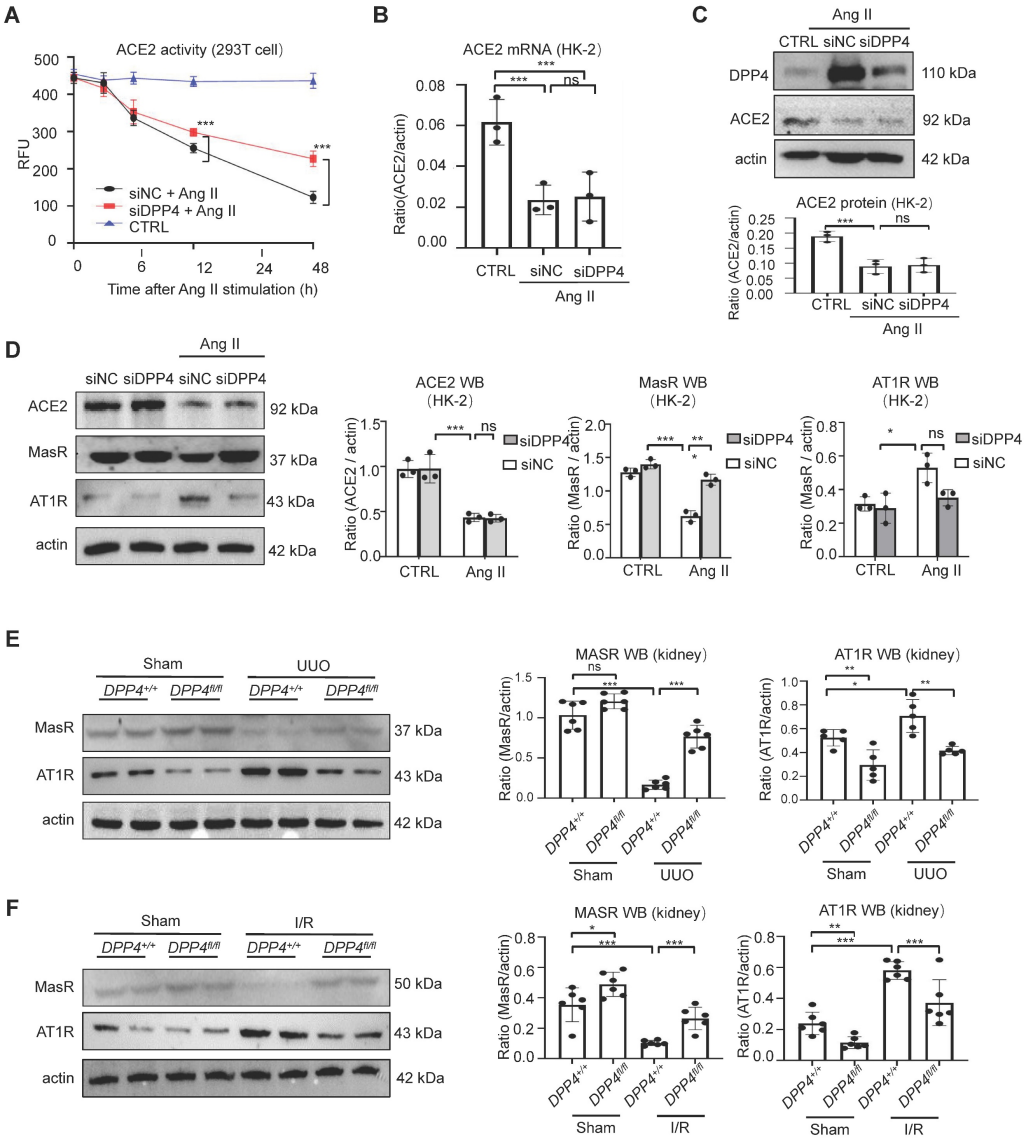
** DPP4 disrupts ACE2/Ang (1-7)/MasR and Ang II/AT1R axis of RAS.** (A) Knockdown of DPP4 could recover ACE2 enzyme activity by using fluorescence resonance energy transfer assay. (B-C) Western blot (B) and real-time PCR (C) show siDPP4-mediated knockdown effectively inhibits DPP4 expression compared to control group (siNC), while no significant alteration is observed in ACE2 expression. (D) Western blot assay and quantification analysis demonstrate that knockdown of DPP4 restores the decrease in Ang II-induced MasR, while alleviating the increase in Ang II-induced AT1R in HK2 cells. (E, F) Western blot assay show MasR and AT1R expression in kidney tissue from UUO (E) and I/R (F) mice. (G) Each bar represents the mean ± SEM of at least 6 mice or at least three independent experiments. *p < 0.05, **p < 0.01, ***p < 0.001. n.s., statistically not significant. siNC, control siRNAs.

DPP4 exerts its biological function through both enzymatic activity and non-enzymatic effects 22. We found a reduction in both protein and mRNA levels of ACE2 following AngII treatment, while inhibition of DPP4 expression failed to restore the expression levels of ACE2. However, restoration of ACE2 enzyme activity was evident (Figure 7A-C). Our findings indicated that ACE2 serves as a substrate of DPP4, and the enzymatic activity of ACE2 is suppressed through its interaction with DPP4 *in vitro*.

Our *in vivo* experiments demonstrated that knockout of DPP4 effectively suppresses fibrosis formation. Interestingly, we found that the expression levels of ACE2 downstream proteins MasR (anti-fibrotic) and AT1R (pro-fibrotic) were significantly altered in the Ang II stimulated HK-2 cells *in vitro* (Figure 7D). Consistently, in the experimental kidney fibrosis models, we observed that the MasR reduction and AT1R induction in the UUO- and I/R-injured kidneys were markedly cancelled in the kidneys of Dpp4 fl/fl; Ksp-Cre mice (Figure 7E-F), uncovering the clinical potential of epithelial DPP4 as a therapeutic target for rebalancing RAS equilibrium in the injured kidney via enhancing ACE2 activity at protein level. Previous studies have shown that blocking AT1R receptors significantly attenuates renal fibrosis. Based on these findings, we conducted the following experiments in HK2 cells: First, we blocked the AT1R receptors, followed by overexpression DPP4 by transfected of GFP-DPP4 plasmid. The results indicated that overexpressing DPP4 significantly exacerbated AngII-induced increases in Fn and α-SMA expression; however, this exacerbation was partially offset when AT1R receptors were blocked (Supplementary Figure 4). Collectedly, these findings suggest that DPP4 promotes renal fibrosis by the ACE2/Ang(1-7)/MasR and Ang II/AT1R axis.

### DPP4 inhibition effectively blocks RAS-mediated renal fibrosis *in vivo*

**Figure 8:**
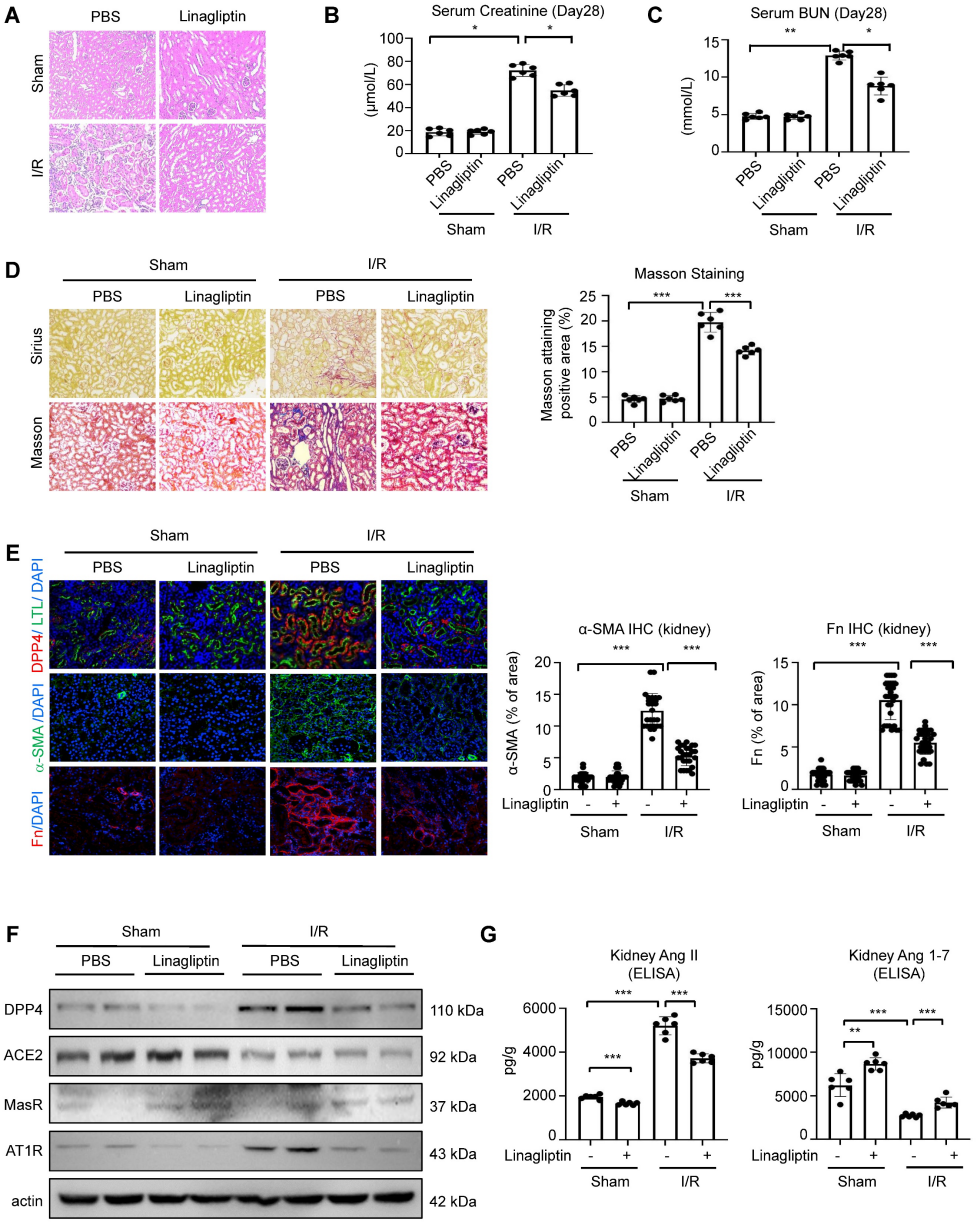
** DPP4 inhibition suppresses renal fibrosis and rebalance resident RAS system in I/R-induced mouse model.** (A) Representative image of hematoxylin and eosin staining of the kidneys. (B, C) Serum creatinine (B) and BUN (C) of the mice in different groups. (D) Representative image showed the Sirius staining and Masson trichrome staining of the kidneys, and quantification analysis of fibrotic area calculated by Masson trichrome staining. (E) Immunofluorescent (IF) analysis of co-staining of DPP4 (Red) and LTL (Green), IF staining of Fn and α-SMA in kidneys. (F) Western blot analysis and quantification analysis of DPP4, ACE2, MasR and AT1R expression in the kidneys. (G) ELISA assay of Ang II (left panel) and Ang 1-7 (right panel) levels of kidneys in each group. n=8. Data were expressed as means ± SEM. *p < 0.05, ***p < 0.001.

To validate the translational potential of our findings, we induced I/R on the wildtype C57 mice and subjected them for a 28-day treatment with FDA approved DPP4 inhibitor linagliptin. Hematoxylin and eosin staining showed that the renal tubular injury induced by I/R injury was attenuated after linagliptin treatment (Figure 8A). Additionally, linagliptin treatment could significantly improve I/R-induced renal function and fibrosis (Figure 8B-8E). Encouragingly, western blot analysis showed that Western blot analysis revealed that linagliptin treatment restored the I/R-induced reduction in MasR expression and inhibited the upregulation of AT1R. Additionally, ELISA assay demonstrated that linagliptin treatment led to a decrease in renal Ang II levels and a recovery of Ang 1-7 levels (Figure 8F-8G).

Our findings clearly demonstrated the translational potential of linagliptin-based DPP4 targeted therapy as an anti-fibrotic strategy for rebalancing RAS signaling via enhancing epithelial specific ACE2/Ang(1-7)/Mas axis and inhibiting Ang II/AT1R axis in kidney patients with high renal Ang II in the clinics.

## Discussion

Renal fibrosis is a prominent feature of chronic kidney disease 23. Despite significant advancements in understanding renal fibrosis; however, effective treatments are still limited because of the complexity of the pathogenic mechanisms. Our previous studies have demonstrated that linagliptin, an FDA-approved specific inhibitor of dipeptidyl peptidase-4 (DPP4), effectively inhibits renal fibrosis in diabetic mice 8. Furthermore, our research and other groups' findings have revealed the abundant expression of DPP4 on the proximal tubule epithelial cells; however, the role of DPP4 in renal fibrosis remains elusive. Therefore, this study aims to elucidate the biological function of kidney-specific DPP4 in renal fibrosis in non-diabetic conditions.

In the past five years, emerging studies and our group have reported that linagliptin exhibits renal protective effects in diabetic nephrotic and other chronic kidney disease 8, 24, 25. The potential mechanism involved the Smad/ERK/P38 and HIF-1α/LOXL2 signaling pathways 24. Additionally, some studies reported that linagliptin may exert its effects directly via GLP-1 receptor (GLP-1R) activation or by modulating oxidative stress pathways in diabetic nephropathy 26. Notably, DPP4 exists in both soluble and membrane-bound forms, which are responsible for proteolytic activity. The soluble form is produced by shedding the membrane-bound DPP4 into circulation to regulate blood glucose levels, while the membrane-bound form exerts pleiotropic actions and is expressed on various cell types, including kidney tubular cells 26. Linagliptin primarily targets DPP4 in the circulation. A limitation of using linagliptin in the research is that it is challenging to distinguish the direct role of DPP4 in kidney disease. Based on our online predictions and immunofluorescence analysis, we found that DPP4 expression was significantly upregulated in the tubular epithelial cells of fibrotic renal tissues. To investigate the biological role of kidney-specific expressed DPP4 rather than circulating DPP4, we constructed epithelial-specific knockout DPP4 (Dpp4fl/fl; Ksp-Cre) mice and established two fibrotic models induced by I/R or UUO injury. Our findings demonstrate that depletion of renal DPP4 effectively restores renal function and suppresses renal fibrosis following I/R or UUO injury compared to the control group. Thus, our study provides the direct evidence demonstrating that high-level, renal tubular-specific expression of DPP4 promotes renal fibrosis.

Mechanically, we identified the promoting role of activated NF-ҡB signaling in the transcriptional regulation of DPP4 expression in the human tubular epithelial cells. It is well established that NF-κB signaling plays a critical role in renal inflammation and fibrosis 27. Our findings indicate that both TGF-β and Ang II, two of the most prominent profibrotic factors, can upregulate DPP4 expression. TGF-β activates NF-κB signaling through crosstalk with the Smad3/Smad7 pathway 28, while Ang II activates NF-κB signaling via the AT1R/ERK/Stat3 or Smad3/Smad7 pathways 28, 29. Interestingly, our research revealed that Ang II has a more significant effect on upregulating DPP4 expression compared to TGF-β-induction, suggesting the potential involvement of additional regulatory mechanisms.

Emerging studies reported that the interaction between DPP4 and ACE2 facilitates the entry of SARS-CoV-2 into cells, suggesting their mutual interaction on the respiratory surface 30. In this study, we observed that highly DPP4 expression unexpectedly enhanced the physical binding with ACE2 on a human proximal tubule epithelial cell line HK-2 by CoIP assay *in vitro*. Numerous studies have highlighted the protective role of ACE2 in various models of renal damage and diseases, including IR-induced kidney injury and UUO-induced kidney injury 31, 32. Emerging studies reported that ACE2 could attenuate the Ang II-induced pressor response, normalize renal Ang II levels, reduce oxidative stress, and prevent Ang II-induced tubulointerstitial fibrosis 33. Our research suggests that by inhibiting local DPP4 expression in the kidney can restore ACE2 activity, and ultimately improve the downstream pathways of ACE2, including the restoration of MasR and the inhibition of AT1R. The inhibition of ACE2 activity leads to reduced degradation of Ang II, which in turn promotes the expression of DPP4 and establishes feedback. This finding suggests that DPP4 may represent an integral component of the RAS system.

Another important finding of this study is the expansion of clinical applications for linagliptin. According to the guidelines of the American Diabetes Association (ADA) and kidney disease: Improving Global Outcomes (KDIGO) 34, linagliptin does not require dose adjustment for patients with CKD, irrespective of the stage of CKD. This forms the fundamental basis for ensuring the safe utilization of linagliptin in patients with CKD. Our results suggest that linagliptin exhibits renoprotective properties in patients with moderate to severe fibrosis, regardless of with diabetic, which establishes a theoretical foundation for future clinical trials into the kidney outcomes.

There are still several limitations in this study, including 1) the lack of direct evidence to demonstrate that the combination of DPP4i and RASi can provide additional therapeutic benefits, and 2) GLP-1 is a known substrate of DPP4 in serum; however, the potential modulation of GLP-1 levels under DPP4 inhibition in the kidney was not evaluated; 3) The regulatory mechanisms of the RAS are complex. Further research is required to elucidate whether DPP4i effect ACE, Ang I, as well as their potential effects on blood pressure 35, 36. Therefore, the roles of DPP4 in kidney disease require further investigations.

Taken together, the present study identified a new pathogenic role of DPP4 in disrupting the balance between Ang(1-7)/MasR and Ang II/AT1R axis by directly binding to ACE2 at the protein level. Targeting of DPP4 may represent a novel therapeutic strategy for renal fibrosis.

## Supplementary Material

Supplementary figures and tables.
