# Supplementary material for: Epithelial DPP4 promotes Ang II-driven renal fibrosis by targeting ACE2 activity in the renin-angiotensin system
Source: Int J Biol Sci. 2025 Jun 9;21(9):3901–16. doi: 10.7150/ijbs.106418 (PMC12210238; doi:10.7150/ijbs.106418)
Supplement: Supplementary file 1 — Supplementary figures and tables. [file ijbsv21p3901s1.pdf]

Supplementary Figure 1

(A) The activation of KEGG pathways was observed in both the mild fibrosis group and the moderate to severe fibrosis group. DPP4 is classified under protein digestion and absorption pathway (highlight in red). (B) *Dpp4* gene expression analysis of a online scRNA-seq analysis from the control adult mice and UUO mice (D14). POD, podocytes; MC, mesangial cells; EC, endothelial cells; PT, proximal tubule; DCT, distal convoluted tubule; CNT, connecting tubule; PCT, proximal convoluted tubule; ICA/B, type A/B intercalated cells; M Φ, macrophage; DL, descending limb; AL, ascending limb; TAL, thick ascending limb; DCT, early distal convoluted tubule; DNT, distal nephron tubule; FIB, fibroblasts.

Supplementary Figure 2

(A) Genotyping of mice. The left panel depicts the homozygous *Dpp4*$^{flox/flox}$ allele, whereas the right panel illustrates the mice carrying the *Cdh16-Cre* (*Ksp-Cre*) phenotype. (B) Immunofluorescent analysis of co-staining of DPP4 (Red) and LTL (Green) in *Dpp4*$^{fl/fl}$*; Ksp-Cre* mice and *Dpp4*$^{+/+}$*; Ksp-Cre* mice with or without unilateral ureteral occlusion (UUO) injure. (C) Immunofluorescent staining of Fn(Red) and α-SMA(Green) in kidneys as indicated groups.

Supplementary Figure 3

(A) Point to point interaction (PPI) of DPP4 and other protein by using conducting a search within the BGI protein Bank. (https://biosys.bgi.com) (B) Immunofluorescent analysis of co-staining of DPP4 (Red) or ACE2 (Red) and LTL (Green) in C57 mice with or without unilateral ureteral occlusion (UUO) injure.

Supplementary Figure 4

Western blot of DPP4, Fn and α-SMA expression in the kidneys with or without transfection of GFP-DPP4 plasmid and cotreat with losartan (10^{-7} M) (an antagonist for type 1 angiotensin receptor) after Ang II treatment.

Supplementary Table 1

siRNA sequence of DPP4 siRNA.

Supplementary Table 2

Sequences of custom-designed primers for quantitative PCR.

Supplementary Table 3

Sequences of truncated plasmid construction.

A

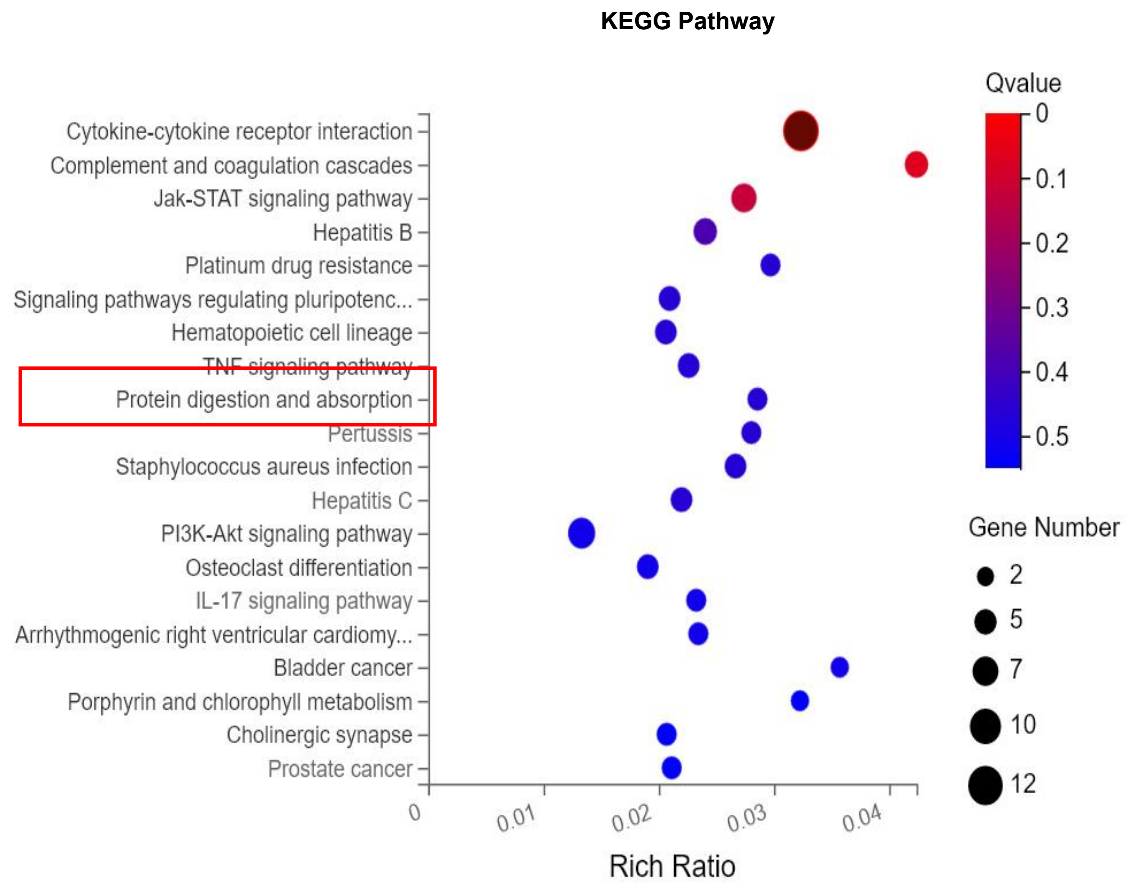

ID	KEGG Pathway Term Desc	Protein
04974	Protein digestion and absorption	DPP4

B

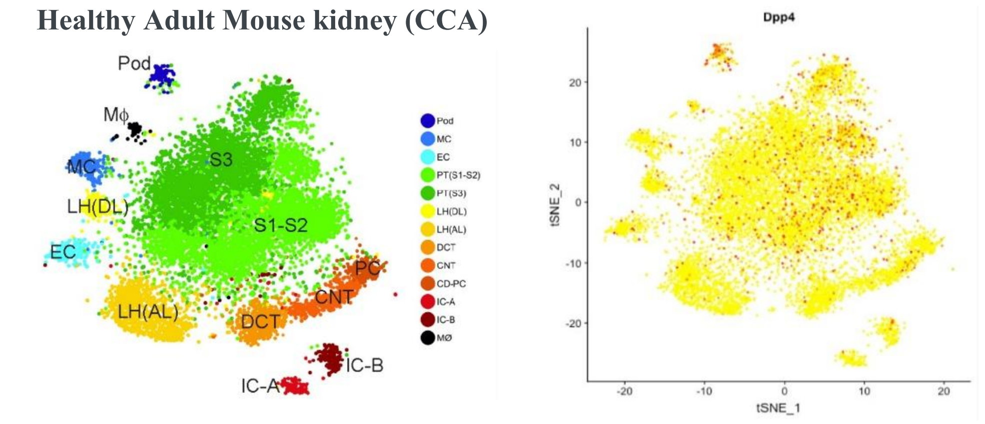

https://humphreyslab.com/SingleCell/

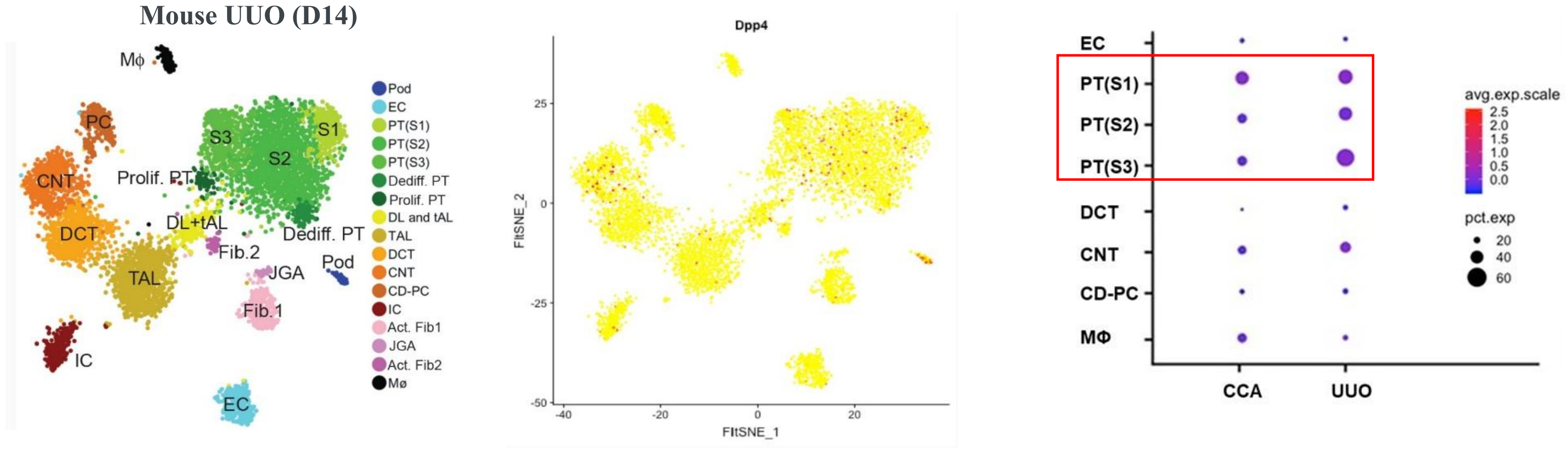

Supplementary Figure 1

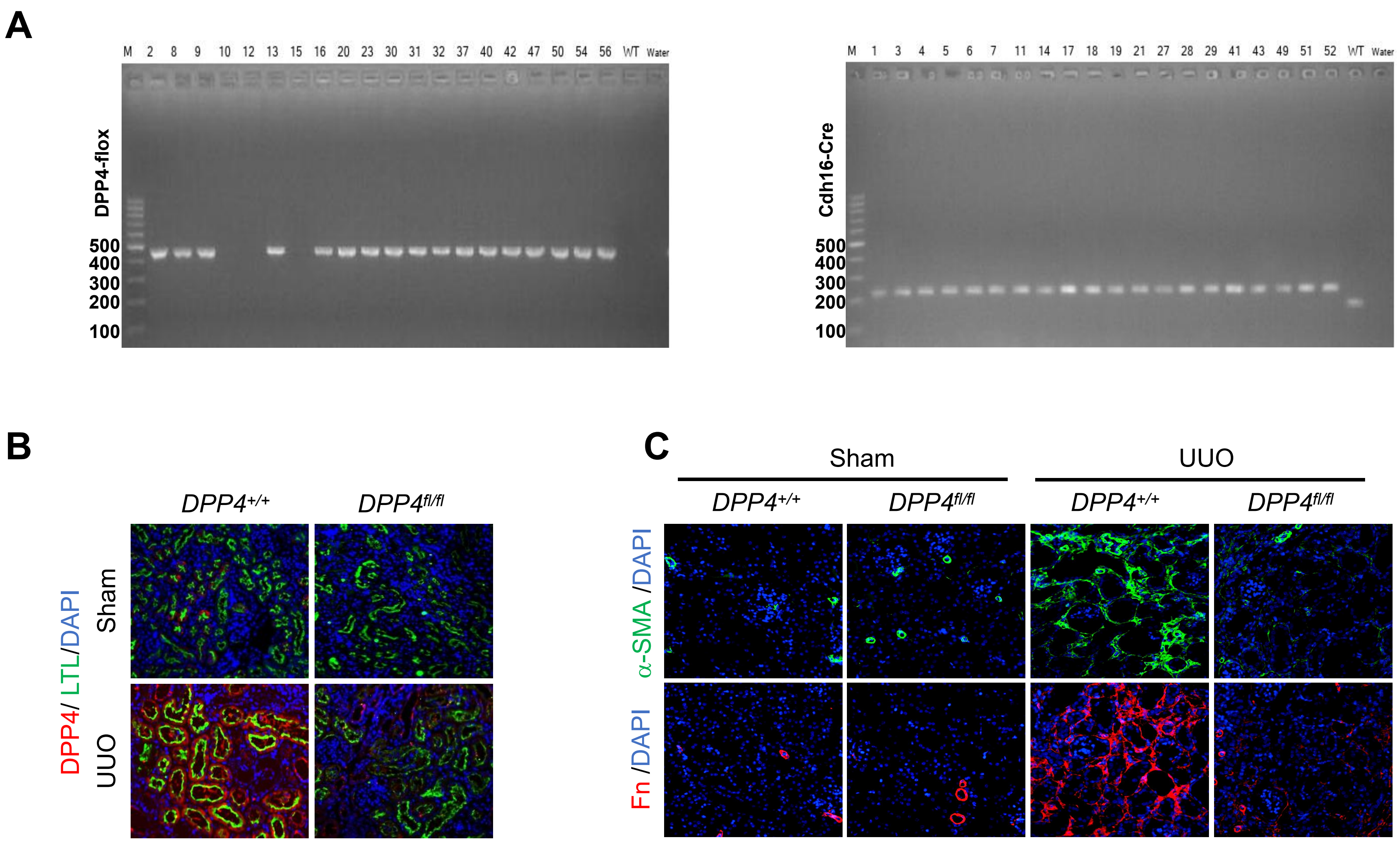

Supplementary Figure 2

A

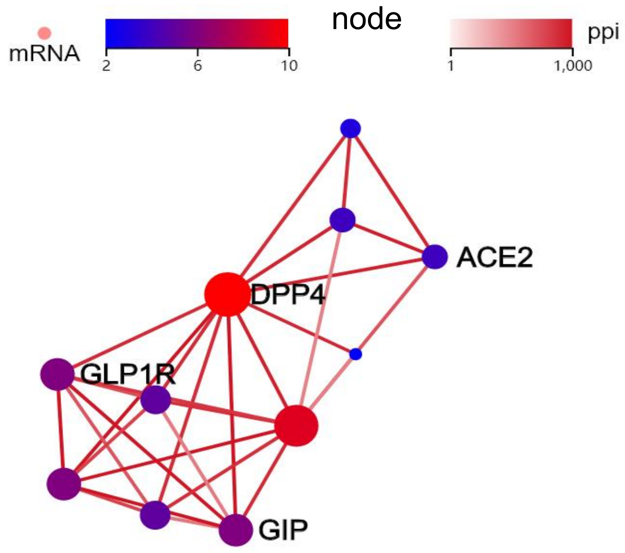

B

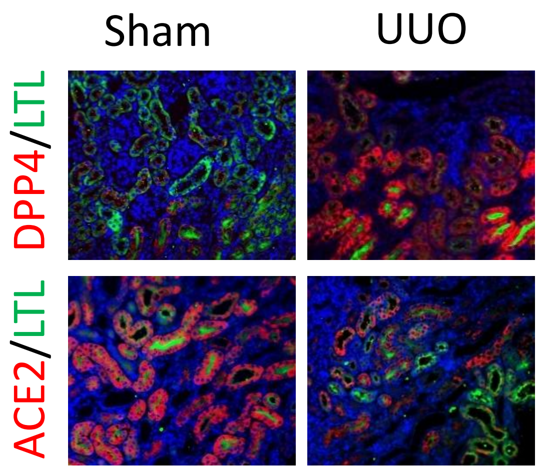

Supplementary Figure 3

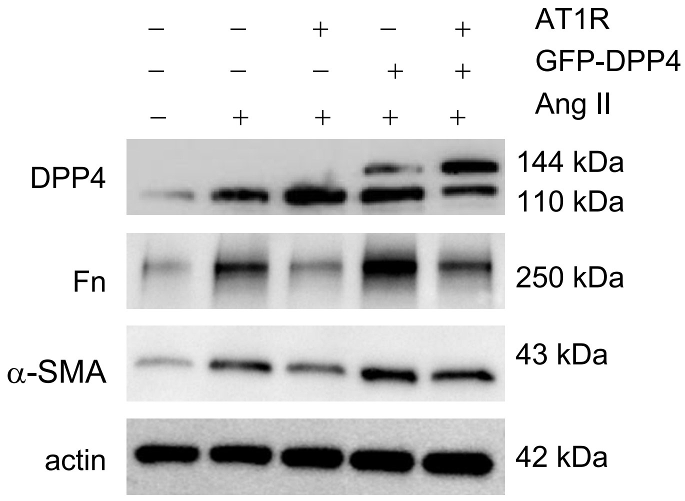

Supplementary Figure 4

siRNA sequence.

Species	siRNA	sequence (5'-3')
Human	DPP4 siRNA	GCAGUACCCAAAGACUGUATT
	Scramble siRNA	UUCUCCGAACGUGUCACGUTT

Supplementary Table 1

Sequences of custom-designed primers for quantitative PCR

Species	Gene	Forward sequence (5' to 3')	Reverse sequence (5' to 3')
Human	DPP4	GCACGGCAACACATTGAA	TGAGGTTCTGAAGGCCTAAATC
Human	ACE2	TGGGATGGAGTACCGACTGGA	GCATATGCAACAGATGATCGGAAC
Mouse	DPP4	TTGTGGATAGCAAGCGAGTTG	CACAGCTATTCCGCACTTGAA
Mouse	ACE2	AATTCAGAATGCGACCATGC	TACTCTTCATATAACGGCCTCAGC
Mouse	MasR	TGACAGCCATCAGTGTGGAGA	GCATGAAAGTGCCCACAGGA
Mouse	AT1R	AACTGCTGGTGTGCCCTACT	AACAGGCCATCTCACTG GTC

Supplementary Table 2

Sequences of truncated plasmid construction

	Gene	Reverse sequence (5' to 3')
DPP4(1-766)	DPP4 FL-F	ATGAAGACACCGTGGAAGGT
	DPP4 FL-R	CTAAGGTAAAGAGAAACATTG
	DPP4 FL-F (HO)	CTATAGGGAGACCCAAGCTTATGAAGACACCGTGGAAGGT
	DPP4 FL-R(HO)	CGGCCGCCAGTGTGATGGATATCCTAAGGTAAAGAGAAACATTG
DPP4(1-492)	DPP4 FL-F	ATGAAGACACCGTGGAAGGT
	DPP4 492-R	CTATCTCAGCCCTTTATCATTCAC
	DPP4 FL-F (HO)	CTATAGGGAGACCCAAGCTTATGAAGACACCGTGGAAGGT
	DPP4 492-R(HO)	GGCCGCCAGTGTGATGGATATCCTATCTCAGCCCTTTATCATTCAC
DPP4(1-290)	DPP4 FL-F	ATGAAGACACCGTGGAAGGT
	DPP4 290 -R	CTAAGGAGCAGTGATTTGTATGG
	DPP4 FL-F (HO)	CTATAGGGAGACCCAAGCTTATGAAGACACCGTGGAAGGT
	DPP4 290-R(HO)	GGCCGCCAGTGTGATGGATATCCTAAGGAGCAGTGATTTGTATGG
DPP4(1-49)	DPP4 FL-F	ATGAAGACACCGTGGAAGGT
	DPP4 49-R	CTATAAGTAATCAGTTAGAGTGT
	DPP4 FL-F (HO)	CTATAGGGAGACCCAAGCTTATGAAGACACCGTGGAAGGT
	DPP4 49-R(HO)	GGCCGCCAGTGTGATGGATATCCTATAAGTAATCAGTTAGAGTGT

HO: Homologous Recombination

Supplementary Table 3
